# Venous Admixture in COPD: Pathophysiology and Therapeutic Approaches

**DOI:** 10.1080/15412550802522783

**Published:** 2009-01-05

**Authors:** Christopher B. Cooper, Bartolome Celli

**Affiliations:** ^1^David Geffen School of Medicine, University of California, Los Angeles, California, USA; ^2^Caritas St Elizabeth's Medical Center, Tufts University, Boston, Massachusetts, USA

**Keywords:** COPD, Gas Exchange, Venous Admixture

## Abstract

Chronic obstructive and interstitial lung diseases impair pulmonary gas exchange leading to wasted ventilation (alveolar dead space) and wasted perfusion (venous admixture). These two fundamental types of abnormality represent opposite ends of the spectrum of ventilation-perfusion mismatch with $\overset{˙}{V}/\overset{˙}{Q}$ ratios of infinity and zero. Treatment approaches that improve airway function, reduce air trapping and hyperinflation have received much attention and might be successful at ameliorating the problems associated with high $\overset{˙}{V}/\overset{˙}{Q}$. However, in patients with low $\overset{˙}{V}/\overset{˙}{Q}$ abnormality in whom venous admixture leads to hypoxemia, there are few therapeutic options. Indeed, some patients are refractory to treatment with supplemental oxygen particularly during exercise. Theoretically these patients could benefit from an intervention that increased mixed venous oxygen content thereby ameliorating the deleterious effects of venous admixture. In this perspective article we discuss the mechanisms whereby venous admixture contributes to hypoxemia and reduced oxygen delivery to tissues. We explore methods which could potentially increase mixed venous oxygen content thus ameliorating the deleterious effects of venous admixture. One such intervention that warrants further investigation is the therapeutic creation of an arterio-venous fistula. Such an approach would be novel, simple and minimally invasive. There is reason to believe that complications would be minor leading to a favorable risk-benefit analysis. This approach to treatment could have significant impact for patients with COPD but should also benefit any patient with chronic hypoxemia that impairs exercise performance.

## INTRODUCTION

Chronic pulmonary diseases, both obstructive and restrictive, impose mechanical constraints on pulmonary ventilation but also disrupt gas exchange through a variety of complex mechanisms (1). Pulmonary gas exchange is crucial for oxygen uptake and carbon dioxide elimination and must be adjusted to match the varying metabolic demands of the body (2).

Gas exchange relies upon efficient matching of ventilation with perfusion in the lungs (3). An ideal ventilation-perfusion ($\overset{˙}{V}/\overset{˙}{Q}$) ratio exists with a numerical value of 0.8 when ventilation and perfusion are expressed in like units (e.g., L/min). $\overset{˙}{V}/\overset{˙}{Q}$ can be considered for the respiratory system as a whole or in terms of many individual lung units each of which could be regarded conceptually as an alveolus and its pulmonary capillaries. An ideal lung would have a uniform distribution of $\overset{˙}{V}/\overset{˙}{Q}$ with values close to 0.8. In reality this does not happen and a spectrum of $\overset{˙}{V}/\overset{˙}{Q}$ exists from zero to infinity. In normal subjects, the main factor that affects $\overset{˙}{V}/\overset{˙}{Q}$ is gravity resulting, in the upright posture, in a gradient from high values at the lung apices to low values at the lung bases.

In patients with chronic pulmonary disease it is the pathological destruction of the lung parenchyma that disrupts $\overset{˙}{V}/\overset{˙}{Q}$ and since the pathology is often unevenly distributed throughout the lungs, the $\overset{˙}{V}/\overset{˙}{Q}$ mismatch is said to be heterogeneous. Certain areas of the lung could be over-ventilated relative to perfusion. In these circumstances $\overset{˙}{V}/\overset{˙}{Q}$ *is high* and a proportion of the ventilation can be considered as “wasted”. Areas of the lung that are ventilated but receive no perfusion have a $\overset{˙}{V}/\overset{˙}{Q}$ ratio of infinity. Other areas of the lung could be under-ventilated relative to perfusion. In these circumstances $\overset{˙}{V}/\overset{˙}{Q}$ *is low* and a proportion of the perfusion can be considered to be “wasted”. Areas of the lung that are perfused but receive no ventilation have a $\overset{˙}{V}/\overset{˙}{Q}$ ratio of zero.

In summary, there are essentially two types of gas exchange abnormality—high $\overset{˙}{V}/\overset{˙}{Q}$ and low $\overset{˙}{V}/\overset{˙}{Q}$. High $\overset{˙}{V}/\overset{˙}{Q}$ increases alveolar dead space thus increasing the ventilatory requirement at a given metabolic rate and affecting the composition of mixed expired gas. Low $\overset{˙}{V}/\overset{˙}{Q}$ results in a phenomenon called venous admixture or physiological shunt, whereby poorly oxygenated blood passes through the lungs and mixes with the blood draining into the left atrium, which will eventually be delivered to the systemic circulation via contraction of the left ventricle (4). The deficit in systemic arterial oxygen content (hypoxemia) which results from venous admixture worsens with increased blood flow through areas of low $\overset{˙}{V}/\overset{˙}{Q}$ as can be seen with increases in cardiac output during exercise.

### Theoretical impact of venous admixture on arterial oxygenation

The impact of venous admixture on arterial oxygenation can be understood by considering the classical equation used to calculate physiological shunt:

$${CaO}_{2}\text{ · }{\overset{˙}{Q}}_{T}\text{ = }C\overset{¯}{\upsilon }O_{2}\text{ · }Q_{S}\text{ + }{Cpc}^{\prime }O_{2}\text{ · }{\overset{˙}{Q}}_{P}$$

where ${\overset{˙}{Q}}_{T}$ is total pulmonary blood flow, ${\overset{˙}{Q}}_{S}$ is pulmonary shunt or venous admixture, ${\overset{˙}{Q}}_{P}$ is the remainder of the pulmonary blood flow and *Cpc′O*_2_ is the end-pulmonary capillary oxygen content.

$$\begin{array}{c} Alternatively\\ {CaO}_{2}=C\overset{¯}{\upsilon }O_{2}\text{ · }\frac{{\overset{˙}{Q}}_{S}}{{\overset{˙}{Q}}_{T}}+{Cpc}^{\prime }O_{2}\text{ · }\frac{{\overset{˙}{Q}}_{P}}{{\overset{˙}{Q}}_{T}}\\ but\;since\\ \frac{{\overset{˙}{Q}}_{P}}{{\overset{˙}{Q}}_{T}}=1-\frac{{\overset{˙}{Q}}_{S}}{{\overset{˙}{Q}}_{T}}\\ then\\ CaO_{2}=C\overset{¯}{\upsilon }O_{2}\frac{{\overset{˙}{Q}}_{S}}{{\overset{˙}{Q}}_{T}}+{Cpc}^{\prime }O_{2}\text{ · }\left(1-\frac{{\overset{˙}{Q}}_{S}}{{\overset{˙}{Q}}_{T}}\right)\\ or\\ {CaO}_{2}=\frac{{\overset{˙}{Q}}_{S}}{{\overset{˙}{Q}}_{T}}(C\overset{¯}{\upsilon }O_{2}-{Cpc}^{\prime }O_{2})+{Cpc}^{\prime }O_{2} \end{array}$$

**Figure 1:**
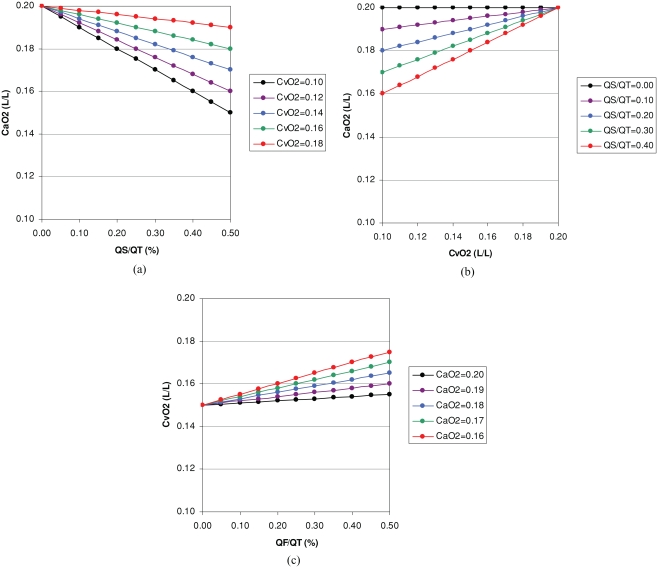
Theoretical calculations based on the classical shunt equation: (a) Effect of venous admixture or pulmonary shunt fraction (${\overset{˙}{Q}}_{S}/{\overset{˙}{Q}}_{T}$) on arterial oxygen content (CaO_2_) for a given mixed venous oxygen content ($C\overset{¯}{v}O_{2}$) assuming ideal pulmonary capillary oxygen content is 0.20 L/L; (b) effect of $C\overset{¯}{v}O_{2}$ on CaO_2_ for a given ${\overset{˙}{Q}}_{S}/{\overset{˙}{Q}}_{T}$ also assuming ideal pulmonary capillary oxygen content is 0.20 L/L; and (c) effect of an arteriovenous stula expressed as a fraction of cardiac output (${\overset{˙}{Q}}_{F}/{\overset{˙}{Q}}_{T}$) on $C\overset{¯}{v}O_{2}$ for a given CaO_2_ assuming systemic capillary oxygen content is 0.15 L/L.

This equation has been used in Figure 1a and 1b to derive *CaO*_2_ for different values of $\frac{{\overset{˙}{Q}}_{S}}{{\overset{˙}{Q}}_{T}}$ and $C\overset{¯}{\upsilon }O_{2}$ assuming that *Cpc′O*_2_ is 0.20 L/L. From these calculations one can deduce that for a pulmonary shunt fraction or venous admixture that equates to 20% of the cardiac output, *CaO*_2_ would decrease by 0.002 L/L (0.2 ml/dl) for a decrease in $C\overset{¯}{\upsilon }O_{2}$ of 0.01 L/L (1 ml/dl).

### The role of oxygenation in exercise limitation

Exercise physiology is a complex subject but practical approaches have increased our understanding (5). With the performance of external work there is an obligatory requirement for energy to facilitate muscle contraction. Ideally, under aerobic conditions, when oxygen is in plentiful supply, increases in external work are match by increases in oxygen uptake. The presence of sufficient oxygen allows complete combustion of metabolic substrate with the generation or regeneration of ATP from oxidative phosphorylation. Hence, it is the contracting muscle that dictates the oxygen uptake required for the performance of exercise (6).

The circulation plays a role in transporting oxygen from the lungs to skeletal muscles and oxygen delivery to tissues can be calculated by the following formula:

DO2=Q˙C · CaO2

where *DO*_2_ is oxygen delivery in L/min, ${\overset{˙}{Q}}_{C}$ is cardiac output also in L/min and *CaO*_2_ is arterial oxygen content in liters of oxygen per liter of blood.

Given the simplicity of this formula it can be seen that ${\overset{˙}{Q}}_{C}$ and *CaO*_2_ are equally important determinants of *DO*_2_. Cardiac output would seem to be important in adjusting oxygen delivery during exercise. However, patients with mild to moderate chronic pulmonary disease do not have difficulty increasing ${\overset{˙}{Q}}_{C}$ on exertion (7) and even patients with severe hyperinflation may have normal hemodynamics (8). On the other hand, patients with severe COPD associated with marked airflow obstruction, hyperinflation and increased work of breathing exhibit large swings in intrathoracic pressure during the breathing cycle that could adversely impact cardiac function (9). Despite these considerations, it seems likely that these patients will have greater difficulty maintaining arterial oxygen content than cardiac output.

The importance of *CaO*_2_ is not simply as a determinant of oxygen delivery by blood flow but also in maintaining an adequate oxygen diffusion gradient in the tissues. At rest, *CaO*_2_ is 0.20 L/L (20 ml/dl) compared with mixed venous oxygen content ($C\overset{¯}{\upsilon }O_{2}$) that is 0.15 L/L (15 ml/dl). Hence, only about 25% of the oxygen available in arterial blood is extracted by the tissues. During exercise *CaO*_2_ does not normally change but $C\overset{¯}{\upsilon }O_{2}$ can fall as low as 0.05 L/L (5 ml/dl) reflecting increased oxygen extraction by exercising muscles. Interestingly, it is not simply the oxygen delivery that is important in providing oxygen to exercising muscle but the fact that a critical oxygen partial pressure can be sustained in the muscle capillaries. This critical capillary oxygen partial pressure (*Pmc′O*_2_) is the necessary driving pressure for oxygen diffusion from the muscle capillary to the mitochondrion. *Pmc′O*_2_ is thought to be approximately 20 mm Hg whereas the mitochondrial oxygen partial pressure is only about 1 mm Hg (10). While the muscle capillary oxygen partial pressure is clearly influenced by oxygen delivery and oxygen utilization in the muscle, it is also crucially dependent on *CaO*_2_. One can easily envisage a situation where ${\overset{˙}{Q}}_{C}$ and muscle blood flow are increased but because of a low *CaO*_2_, the *Pc′O*_2_ cannot be maintained.

Let us then consider the pathologies that affect the provision of oxygen to skeletal muscle. Firstly, there is cardiac failure resulting in low ${\overset{˙}{Q}}_{C}$. *DO*_2_ is reduced in direct proportion to the reduced ${\overset{˙}{Q}}_{C}$; however, typically, in this setting, tissue oxygen extraction proceeds resulting in a lower $C\overset{¯}{\upsilon }O_{2}$. Provided *CaO*_2_ is adequate, and ${\overset{˙}{Q}}_{C}$ is not extremely low, it should still be possible to maintain the *Pmc′ O*_2_.

Hemoglobin concentration is another factor which influences *CaO*_2_ because *CaO*_2_ is calculated as the sum of the oxygen combined with hemoglobin and that dissolved in the plasma.

The formula that determines *CaO*_2_ is as follows (11):

CaO2=[H b] · 1.34 · SaO2+PaO2 · 0.0031

where *CaO*_2_ is arterial oxygen content in ml of oxygen per deciliter of blood, *SaO*_2_ is the percentage oxyhemoglobin saturation, [*Hb*] is hemoglobin concentration in g/dl, *PaO*_2_ is the arterial oxygen partial pressure in mm Hg.

This formula shows that in anemia, a reduction of hemoglobin concentration of 1 g/dl will result in approximate 6–7% reduction in arterial oxygen content. The importance of this observation in patients with chronic pulmonary disease is unknown but many of these patients have anemia or relative anemia (12) and anemia adversely affects exercise capacity in COPD (13).

Venous admixture is arguably a much more serious consideration in terms of providing oxygen to skeletal muscle. To the extent that venous admixture lowers *CaO*_2_ it will also reduce the driving pressure for oxygen diffusion in the tissues and in these circumstances it might not be possible to maintain the *Pmc′O*_2_. Interestingly, in circumstances of skeletal muscle dysfunction where the muscle is unable to utilize oxygen because of structural or metabolic abnormalities, then $C\overset{¯}{\upsilon }O_{2}$ might actually be higher than normal (14).

Hence, the complex pathophysiology which occurs in chronic pulmonary diseases can impair exercise performance in several ways. Increased ventilatory requirement for a given level of exercise, causes dyspnea and, in some cases, ventilatory limitation (15). Hypoxemia reduces oxygen delivery to skeletal muscles as well as markedly worsening dyspnea through carotid body stimulation.

### Approaches to improving exercise performance in chronic obstructive pulmonary disease

Chronic obstructive pulmonary disease (COPD) is a progressive disease that encompasses chronic bronchitis and emphysema (16). COPD is the most common chronic pulmonary disease worldwide and is thought to affect 24 million Americans (17). About 2–3 million of these are likely to have severe disease with significant hypoxemia and exercise impairment. The mechanisms of exercise limitation described above are highly relevant to patients with severe COPD. They have reduced aerobic capacity, deconditioning, increased ventilatory requirement and chronic hypoxemia that predictably worsens with exertion (18). When these patients reach this stage of disease severity their therapeutic options are limited. Nevertheless, there has been considerable progress in recent years in developing approaches to improve exercise performance in patients with chronic pulmonary diseases, particularly in COPD (19).

Inhaled bronchodilator therapy is regarded as important in the maintenance treatment of COPD because of the demonstrated benefits on dyspnea, exercise performance and quality of life. Studies of long-acting bronchodilators indicate that these benefits relate to reduced static and dynamic hyperinflation (20–22). Phenotypically, therefore, the type of patient who benefits from bronchodilator therapy, at least in terms of reducing lung volume, has emphysematous destruction of the lung parenchyma and a component of high $\overset{˙}{V}/\overset{˙}{Q}$ abnormality.

Even in patients with severe chronic pulmonary diseases, physical reconditioning can be achieved through structured exercise training programs (23). These programs reduce lactic acid accumulation and hence reduce ventilatory requirement for a given level of exercise (24). They also improve breathing efficiency, regardless of the reconditioning effect and this benefit probably relates to slowed breathing frequency (25).

Patients who demonstrate hypoxemia during exercise show improved exercise performance with supplemental oxygen (26). An obvious mechanism for this benefit would be correction of the hypoxemia but a secondary improvement in breathing mechanics might also occur due to reduced breathing frequency from suppression of carotid body activity (27).

**Figure 2:**
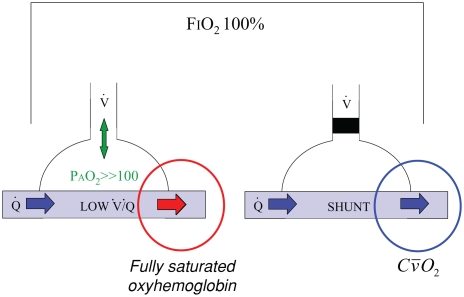
Effect of supplemental oxygen on ventilation-perfusion mismatch (low $\overset{˙}{V}/\overset{˙}{Q}$) and intra-pulmonary shunt ($\overset{˙}{V}/\overset{˙}{Q}=0$). When breathing 100% oxygen from a closed circuit (FIO_2_ 100%), even lung regions with very low alveolar ventilation (on the left) should see an increase in alveolar oxygen tension (PAO2) to greater then 100 mm Hg in which case the blood draining that region will be fully saturated. By contrast, regions of intra-pulmonary shunt (on the right) experience no ventilation and the blood draining those regions has an oxygen content equal to the mixed venous blood ($C\overset{¯}{v}O_{2}$).

Supplemental oxygen is considered effective at correcting the hypoxemia that results from $\overset{˙}{V}/\overset{˙}{Q}$ mismatch in the lungs. The rationale is shown in Figure 2.

By substantially increasing the inspired oxygen concentration and partial pressure, it is believed that even areas of low $\overset{˙}{V}/\overset{˙}{Q}$ that have low levels of ventilation will experience a sufficient increase in alveolar oxygen partial pressure (> 100 mm Hg) to fully oxygenate the blood in the adjacent pulmonary capillaries. This will reduce but not abolish venous admixture because areas with no ventilation ($\overset{˙}{V}/\overset{˙}{Q}=0$) will not be influenced by the inspired oxygen concentration.

To the extent that a patient with chronic pulmonary disease has increased physiological shunt, that patient will be refractory to the benefits from supplemental oxygen. These pathophysio-logical effects are seen in many types of lung disease but particularly in patients with COPD (18) and pulmonary fibrosis (28).

### Mechanisms to reduce the adverse consequences of venous admixture

Venous admixture becomes extremely relevant as a cause of hypoxemia in patients with chronic pulmonary diseases, particularly during exercise when cardiac output is increased and flow through areas of intra-pulmonary shunt is increased. Furthermore, as noted above, supplemental oxygen only benefits areas of $\overset{˙}{V}/\overset{˙}{Q}$ mismatch and patients with extensive intra-pulmonary shunt may actually be refractory to this treatment. Elevation of $C\overset{¯}{\upsilon }O_{2}$ should ameliorate the deleterious effects of venous admixture both at rest and also, importantly during exercise. In therapeutic terms there are few ways to increase $C\overset{¯}{\upsilon }O_{2}$.

One simple method would be to increase [*Hb*] by blood transfusion or stimulation of erythropoesis. Autotransfusion of blood in athletes (blood doping) is well know to enhance aerobic performance although in the context of an athlete the mechanisms are probably somewhat different (29). Improved exercise performance related to increased red cell mass can also achieved in athletes by stimulation of erythropoesis with hypobaric hypoxia (altitude) (30). Human erythropoietin or its analogues have been considered but concerns exist as to what is an appropriate [*Hb*] in a patient with chronic hypoxemia and what is the risk of intravascular thrombosis due to increased blood viscosity (31). The cost of these agents is also likely to be a limiting factor.

A more intriguing method by which to increase $C\overset{¯}{\upsilon }O_{2}$ is the therapeutic creation of an arterio-venous (AV) fistula. This approach would shunt oxygenated arterial blood from the systemic arterial circulation to the systemic veins thus directly influencing and increasing $C\overset{¯}{\upsilon }O_{2}$. There has been extensive previous experience of artificially created AV fistulae in the context of renal failure patients needing hemodialysis (32). A theoretical advantage of the therapeutic AV fistula is that it should improve hypoxemia not only in patients with $\overset{˙}{V}/\overset{˙}{Q}$ mismatch but also those with increased physiological shunt who are refractory to treatment with supplemental oxygen.

The effect of an arteriovenous fistula on $C\overset{¯}{\upsilon }O_{2}$ can be explored theoretically with an equation analogous to that used for the calculation of *CaO*_2_ taking into account physiological shunt:

$$C\overset{¯}{\upsilon }O_{2}\cdot {\overset{˙}{Q}}_{T}={CaO}_{2}\cdot Q_{F}+{Csc}^{\prime }O_{2}\cdot {\overset{˙}{Q}}_{NF}$$

where ${\overset{˙}{Q}}_{T}$ is total systemic blood flow, ${\overset{˙}{Q}}_{F}$ is the blood flow through the fistula, ${\overset{˙}{Q}}_{NF}$ is the remainder of the systemic blood flow and *Csc′O*_2_ is the mixed systemic capillary oxygen content.

$$\begin{array}{c} Alternatively\\ C\overset{¯}{\upsilon }O_{2}={CaO}_{2}\cdot \frac{{\overset{˙}{Q}}_{F}}{{\overset{˙}{Q}}_{T}}+{Csc}^{\prime }O_{2}\cdot \frac{{\overset{˙}{Q}}_{NF}}{{\overset{˙}{Q}}_{T}}\\ but\;since\\ \frac{{\overset{˙}{Q}}_{NF}}{{\overset{˙}{Q}}_{T}}=1-\frac{{\overset{˙}{Q}}_{F}}{{\overset{˙}{Q}}_{T}}\\ then\\ C\overset{¯}{\upsilon }O_{2}={CaO}_{2}\cdot \frac{{\overset{˙}{Q}}_{F}}{{\overset{˙}{Q}}_{T}}+{Csc}^{\prime }O_{2}\cdot \left(1-\frac{{\overset{˙}{Q}}_{F}}{{\overset{˙}{Q}}_{T}}\right)\\ or\\ C\overset{¯}{\upsilon }O_{2}=\frac{{\overset{˙}{Q}}_{F}}{{\overset{˙}{Q}}_{T}}({CaO}_{2}-{Csc}^{\prime }O_{2})+{Csc}^{\prime }O_{2} \end{array}$$

This equation has been used in Figure 1c to derive $C\overset{¯}{\upsilon }O_{2}$ for different values of $\frac{{\overset{˙}{Q}}_{F}}{{\overset{˙}{Q}}_{T}}$ and *CaO*_2_ assuming that *Csc′O*_2_ is 0.15 L/L. From these calculations one can deduce that for a *CaO*_2_ of 0.18 L/L (18 ml/dl or ≈90% saturation), an increase in flow through the fistula of 0.1 (10% of total systemic blood flow) would increase $C\overset{¯}{\upsilon }O_{2}$ by 0.003 L/L (0.3 ml/dL).

Any COPD patient with hypoxemia, either chronically at rest or intermittently during exercise, would be expected to have a range of low $\overset{˙}{V}/\overset{˙}{Q}$ abnormalities in the lungs. In these patients, the component of “$\overset{˙}{V}/\overset{˙}{Q}$ mismatch” or low $\overset{˙}{V}/\overset{˙}{Q}$ would be expected to improve, at least to some extent, with supplemental oxygen. The same patients would most likely have a degree of venous admixture which would be refractory to supplemental oxygen but could potentially be improved by creation of an arterio-venous fistula that elevates $C\overset{¯}{\upsilon }O_{2}$.

### Potential adverse consequences of an arterio-venous fistula

Peripheral arterio-venous fistulae or shunts represent a reduction in systemic vascular resistance and therefore tend to be associated with an increased cardiac output. The question obviously arises as to how this would be tolerated over time. Furthermore, patients with chronic pulmonary disease might already be experiencing right ventricular compromise from increased afterload due to hypoxic pulmonary vasoconstriction or pulmonary vascular remodeling. An increased ${\overset{˙}{Q}}_{C}$ could worsen this situation and lead to further increases in pulmonary arterial pressures. On the other hand, if the elevation of $C\overset{¯}{\upsilon }O_{2}$ contributed to an improvement of oxygenation within the lung there could be a reduction in hypoxic pulmonary vasoconstriction, reduction in pulmonary vascular resistance and paradoxical reduction in pulmonary arterial pressures despite the increased ${\overset{˙}{Q}}_{C}$. Preliminary data from patients with severe COPD suggest that these effects can occur (33).

Another concern would be that creation of an AV fistula would divert some of the arterial blood flow to that limb resulting in a relative deprivation of oxygen delivery. Whilst theoretically possible, it is also likely that local autoregulation would preserve the arterial blood flow distal to the fistula and that the increased ${\overset{˙}{Q}}_{C}$ is in part a reflection of this adaptation.

## SUMMARY

Patients with chronic pulmonary disease that have disrupted gas exchange which increases physiological shunt and venous admixture. These patients have serious problems with oxygenation and are often somewhat refractory to treatment with supplemental oxygen particularly during exercise. Theoretically they could benefit from an intervention that increased mixed venous oxygen content thereby ameliorating the deleterious effects of venous admixture.

One such intervention that warrants further investigation is the therapeutic creation of an arterio-venous fistula. Such an approach would be novel, simple and minimally invasive. There is reason to believe that complications would be minor leading to a favorable risk-benefit analysis. This approach to treatment could have significant impact for patients with COPD but should also benefit any patient with chronic hypoxemia that impairs exercise performance.
